# Crosses Heterozygous for Hybrid *Neurospora* Translocation Strains Show Transmission Ratio Distortion Disfavoring Homokaryotic Ascospores Made Following Alternate Segregation

**DOI:** 10.1534/g3.116.030627

**Published:** 2016-06-17

**Authors:** Dev Ashish Giri, Selvam Rekha, Durgadas P. Kasbekar

**Affiliations:** * Centre for DNA Fingerprinting and Diagnostics, Hyderabad 500001, India; † Graduate Studies, Manipal University, Karnataka 576104, India

**Keywords:** chromosome translocation, introgression, segregation distortion, meiotic drive

## Abstract

By introgressing *Neurospora crassa* translocations into *N. tetrasperma*, we constructed heterokaryons bearing haploid nuclei of opposite mating types, and either the translocation and normal sequence chromosomes (*i.e.*, [*T* + *N*]) or a duplication and its complementary deficiency (*i.e.*, [*Dp* + *Df*]). The [*T* + *N*] heterokaryons result from alternate segregation of homologous centromeres, whereas adjacent-1 segregation generates [*Dp* + *Df*]. Self-cross of either heterokaryon produces [*T* + *N*] and [*Dp* + *Df*] progeny. Occasionally during *N. tetrasperma* ascus development, a pair of smaller homokaryotic ascospores replaces a heterokaryotic ascospore. Crosses with the *Eight-spore* mutant increase such replacement, and can generate asci with eight homokaryotic ascospores, either 4*T* + 4*N* from alternate segregation, or 4*Dp* + 4*Df* from adjacent-1 segregation. Crosses of some of the introgressed translocation strains with normal sequence *N. tetrasperma* produced more *Dp* than *T* or *N* homokaryotic progeny. We suggest this is due to an insufficiency for a presumptive ascospore maturation factor, which increases the chance that, in asci with > 4 viable ascospores, none properly mature. Since only four viable ascospores (*Dp* or [*Dp* + *Df*]) share the limiting factor following adjacent-1 segregation, whereas four to eight ascospores compete for it following alternate segregation, this would explain why *Dp* homokaryons outnumber *T* and *N* types, whereas the heterokaryons are not as affected. We believe that this novel form of transmission ratio distortion is caused by a Bateson–Dobzhansky–Muller Incompatibility (BDMI) triggered by an *N. crassa* gene in the *N. tetrasperma* background. Heterokaryons tend not to out-cross, and crosses of *Dp* strains are barren, thus the BDMI impedes interspecies gene flow.

*Neurospora crassa* and *N. tetrasperma* are related fungal species but they differ strikingly in ascus development. *N. crassa* asci form eight initially uninucleate ascospores, whereas *N. tetrasperma* asci make four initially binucleate ascospores (Figure 1). *N. crassa* ascospores are homokaryons of either *mat A* or *mat a* type, and produce mycelia that can mate with mycelia of the opposite mating type derived from another ascospore. *N. tetrasperma* ascospores are [*mat A* + *mat a*] dikaryons, and produce mycelia that can undergo a self-cross. Occasionally, a pair of smaller homokaryotic ascospores can replace one or more dikaryotic ascospores (Raju 1992; Raju and Perkins 1994). The dominant *Eight-spore* (*E*) mutant increases such replacement, and can generate asci with eight homokaryotic ascospores (Dodge 1939; Calhoun and Howe 1968). *N. tetrasperma* dikaryotic mycelia also produce some homokaryotic conidia (vegetative spores) by chance, and mycelia from homokaryotic conidia and ascospores can out-cross with like mycelia of the opposite mating type (Raju and Perkins 1994; Bistis 1996). Ascus development in *N. crassa* lends itself to the detection and characterization of chromosome rearrangements, and 355 chromosome rearrangements were described in *N. crassa* (Perkins 1997), whereas *N. tetrasperma* ascus development masks the presence of rearranged chromosomes, and no translocation chromosomes have, thus far, been reported in this species. Recently, we showed that introgression of insertional and quasi-terminal translocations from *N. crassa* into *N. tetrasperma* allows us to generate novel heterokaryotic strains with complementary duplications and deficiencies ([*Dp* + *Df*]) in their constituent nuclei (Giri *et al.* 2015). Such heterokaryons have never been made previously in any species. The introgressed translocation strains, designated as *T^Nt^*, nominally have a *N. tetrasperma* genome, except at the *N. crassa*-derived translocation breakpoint junctions. The crosses that yielded [*Dp* + *Df*] heterokaryons also produced [*T^Nt^* + *N*] heterokaryons, whose constituent nuclei have the translocation (*T^Nt^*) and normal sequence (*N*) chromosomes. [*Dp* + *Df*] and [*T^Nt^* + *N*] heterokaryons share identical genes and hence are expected to share identical phenotypes. Were they, however, to display different phenotypes, it might flag the absence of one or more ‘nucleus-limited’ genes from the *Df* nuclei (Kasbekar 2014; Giri *et al.* 2015). That is, genes for which nuclei bearing the null allele (Δ) fail to be complemented by neighboring wild-type nuclei (*WT*) in [*WT* + Δ] heterokaryons (Kasbekar 2014).

**Figure 1:**
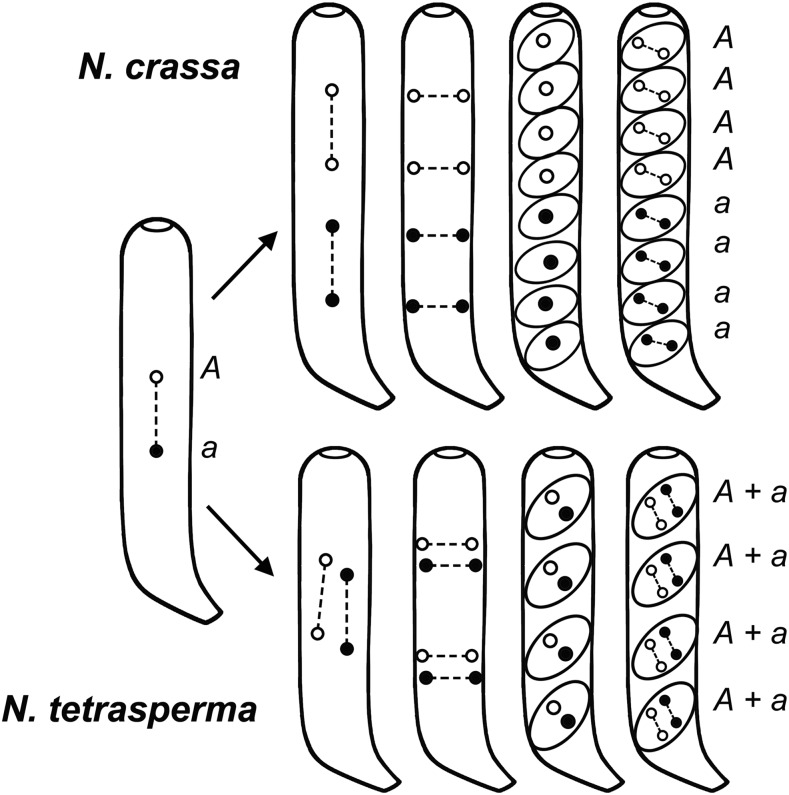
Ascus development in *N. crassa* and *N. tetrasperma*. Fusion of the parental haploid *mat A* and *mat a* nuclei (respectively, open and filled circles) produces a diploid zygote nucleus that undergoes meiosis (leftmost panel shows meiosis I, the *mat A* and *mat a* loci show first division segregation) and a postmeiotic mitosis (third panels from left) to generate eight haploid progeny nuclei (4 *mat A* + 4 *mat a*). In *N. crassa* (upper panels), these nuclei are partitioned into eight initially uninucleate ascospores formed per ascus, whereas in *N. tetrasperma* (lower panels) the asci make four initially binucleate ascospores, each receiving a pair of nonsister nuclei (1 *mat A* + 1 *mat a*). *N. crassa* ascospores produce homokaryotic mycelia of *mat A* or *mat a* type that can mate only with mycelia derived from another ascospore of the opposite mating type. In contrast, dikaryotic [*mat A* + *mat a*] *N. tetrasperma* mycelia can undergo a self-cross. Figure adapted from Figure 4 of Raju and Perkins (1991).

An insertional translocation transfers a donor chromosome segment into a recipient chromosome without any reciprocal exchange (Perkins 1997). It creates three breakpoint junctions: A on the donor chromosome, and B and C (proximal and distal) on the recipient chromosome (Figure 2). A quasi-terminal translocation transfers a distal segment of the donor chromosome to the tip of a recipient chromosome, distal to any essential gene, and presumably the recipient chromosome’s tip caps the donor chromosome’s break. A reciprocal translocation reciprocally interchanges the terminal segments of two chromosomes. Quasi-terminal and reciprocal translocations create two breakpoint junctions (Supplemental Material, Figure S1, A and B). We have determined the breakpoint junctions of several insertional, quasi-terminal, and reciprocal translocations (Singh 2010; Singh *et al.* 2010).

**Figure 2:**
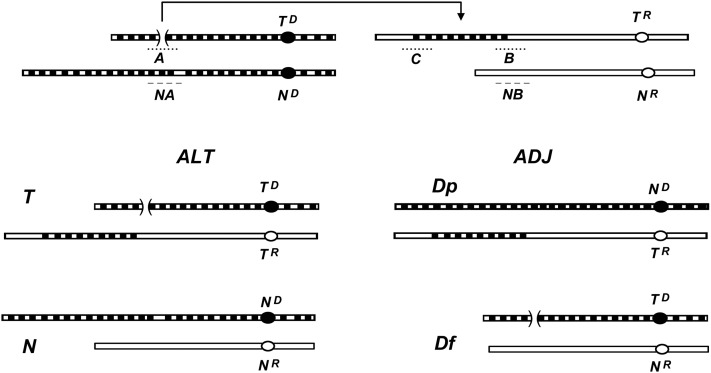
Alternate (ALT) and adjacent-1 (ADJ) segregation in a normal sequence (*N*) by insertional translocation (*IT*) cross. *T^D^* and *T^R^* designate the donor and recipient chromosomes of the *IT*, and *N^D^* and *N^R^* are their *N*-derived homologs. The A, B, and C breakpoint junctions are indicated by the dotted lines, and dashed lines NA and NB indicate segments in the normal sequence homologs that are disrupted in the translocation chromosomes. In ALT (lower left), *T^D^* and *T^R^* segregate to one spindle pole, and *N^D^* and *N^R^* to the other. Subsequently, meiosis II and postmeiotic mitosis generate eight parental-type nuclei, *viz*. 4 *T* + 4 *N*. In ADJ (lower right), *N^D^* and *T^R^* segregate to one pole and *T^D^* and *N^R^* to the other, to eventually produce eight nonparental nuclei, 4 *Dp* + 4 *Df*. The *T*, *N*, and *Dp* types are viable, whereas the *Df* type is inviable. *T* progeny contain the A, B, and C breakpoints, *Dp* contain B and C but not A, and *N* contain none.

In crosses heterozygous for a nonreciprocal translocation, segregation of homologous centromeres has one of two outcomes. The subsequent haploid chromosome complements may be balanced if segregation is “alternate” (ALT), having either both normal sequence or both translocation chromosomes, or unbalanced if segregation is adjacent-1 (ADJ), where one has a duplication and the other the complementary deficiency (Figure 2). In *N. crassa*, ALT produces eight black (B), viable parental ascospores, while ADJ gives eight nonparental ascospores, with the four duplication spores being black and viable, and the four deficiency spores white (W) and nonviable. Since either segregation is equally likely, asci with 8B:0W and 4B:4W ascospores occur in equal numbers. Additionally, some asci can have 6B:2W ascospores as a result of recombination in the interstitial regions between the centromeres and breakpoints, but asci with 2B:6W and 0B:8W ascospores are not expected (Perkins 1997). ALT segregation for *N. crassa* reciprocal translocation heterozygotes also gives 8B:0W asci. ADJ segregation, which yields a mix of translocation and normal sequence chromosomes, results in deficiencies in every spore (Figure S1). Thus, equal numbers of asci with 8B:0W and 0B:8W ascospores characterize heterozygosity for a reciprocal translocation. Most asci from isosequential crosses (*i.e.*, *N* × *N* or *T* × *T*) have 8B:0W ascospores (Perkins 1997).

In *N. tetrasperma*, crosses heterozygous for a nonreciprocal translocation ALT and ADJ generate 4B:0W asci that contain, respectively, [*T^Nt^* + *N*] and [*Dp* + *Df*] heterokaryotic ascospores. The constituent nuclei in these ascospores are of opposite mating type (*i.e.*, [*mat a* + *mat A*]) and, hence, the resulting mycelia can undergo a self-cross. The [*T^Nt^* + *N*] and [*Dp* + *Df*] genotypes can interconvert through self-crosses (Giri *et al.* 2015).

We had introgressed four insertional translocations (*viz*. EB4, IBj5, UK14-1, and B362i) from *N. crassa* to *N. tetrasperma*, and the introgressed translocation strains were designated as *T(EB4)^Nt^*; *T(IBj5)^Nt^*; *T(UK14-1)^Nt^* and *T(B362i)^Nt^* (Giri *et al.* 2015). Crosses of the *T^Nt^* strains with the standard laboratory *N. tetrasperma* strains 85 *A* or 85 *a* produced mostly four-spored asci, but the rare eight-spored asci included ones with 8B:0W, 4B:4W, and 6B:2W ascospores (Giri *et al.* 2015). The number of eight-spored asci was increased when the *T^Nt^* strains were crossed with the *E* mutant strains of opposite mating type. As expected, the crosses of *T(EB4)^Nt^* and *T(UK14-1)^Nt^* with *E* yielded comparable numbers of asci with 8B:0W and 4B:4W ascospores, and also some with 6B:2W ascospores. Surprisingly, the crosses of *T(IBj5)^Nt^* and *T(B362i)^Nt^* with *E* did not yield any asci with 8B:0W and 6B:2W ascospores (Giri *et al.* 2015). Since no ascus contained more than four black ascospores, we designate this as the max-4^-^ phenotype. Previous data suggested that a recessive mutation derived from strain 343.6 *A E*^-^ was present in the *E*, *T(IBj5)^Nt^*, and *T(B362i)^Nt^* strains, and blocked ascospore maturation in eight-spored asci formed following ALT, but not in asci formed following ADJ (Giri *et al.* 2015). We now show that this model is not tenable. Instead, we suggest that a Bateson–Dobzhansky–Muller Incompatibility (BDMI) between *N. crassa* and *N. tetrasperma* genes is responsible. In BDMI, the alleles of one species are unable to function well with alleles at another locus from a closely related species, thus causing inviability or reduced fertility in the interspecies hybrids (Orr *et al.* 2007; Fishman and Saunders 2008; McDermott and Noor 2010; Phadnis 2011; Presgraves 2010; Zanders *et al.* 2014; Phadnis *et al.* 2015; Sicard *et al.* 2015). We suggest that a BDMI between the two species can block ascospore maturation in asci bearing more than four viable ascospores, which reduces the survival of progeny with a balanced translocation. If the BDMI kicks in before the introgression of *N. crassa* sequences into *N. tetrasperma* advances sufficiently to produce self-fertile heterokaryons, the introgression effort may be unsuccessful.

## Materials and methods

### Neurospora strains and general genetic manipulations

*Neurospora* genetic analysis was done essentially as described by Davis and De Serres (1970). The alternative recipe of Metzenberg (2003) was used to make Medium N. Table S1 lists the *Neurospora* strains used. They were obtained from the Fungal Genetics Stock Center (FGSC, Department of Plant Pathology, Kansas State University).

The *N. crassa*/*N. tetrasperma* hybrid strain *C4,T4 a* was used as a bridging strain in the introgression crosses. It has four *N. crassa* great-grandparents and four *N. tetrasperma* great-grandparents. The *N. crassa* great-grandparents were of the OR background, whereas the *N. tetrasperma* great-grandparents were of the 343.6 *A E* background (Metzenberg and Ahlgren 1969).

Crosses of *T^Nt^* strains with *N. tetrasperma* strain 85 derivatives of opposite mating type (*T^Nt^* × 85) yield self-fertile heterokaryotic progeny of [*T^Nt^* + *N*] or [*Dp* + *Df*] genotype. [*T^Nt^* + *N*] mycelia yield homokaryotic (self-sterile) conidial derivatives of both mating types, whereas [*Dp* + *Df*] mycelia yield homokaryons of only the mating type of the *Dp* nucleus. The *T^Nt^* strains are reisolatable as self-sterile conidial derivatives from the [*T^Nt^* + *N*] mycelia.

### Markers polymorphic between the 85/EA/Ea and FGSC 2508A/FGSC 2509a strains

The genome sequence of the *N. tetrasperma* strain FGSC 2508 *A* and FGSC 2509 *a* is publicly available (fungidb.org). The *N. tetrasperma* strains 85, *EA*, and *Ea* share considerable DNA sequence homology (data not shown), and their genome is more diverged from that of FGSC 2508*A* and FGSC 2509*a*. Table S2 lists the oligonucleotide primers used for PCR and restriction enzymes used to digest the resulting amplicons to obtain polymorphisms between the *85*/*EA*/*Ea* and FGSC 2508 *A*/FGSC 2509 *a* alleles on each of the seven *N. tetrasperma* chromosomes. Figure S2 gives the positions of the marker on the chromosomes.

### PCR-based identification of T, N, and Dp progeny from T^Nt^ × N crosses

*IT*s are defined by breakpoint junction A on the donor chromosome, and junctions B and C (proximal and distal) on the recipient chromosome, whereas *QT*s have only two breakpoint junctions, A and B, on the two participating chromosomes. Additionally, primers to PCR amplify genome segments from the *N*-derived donor and recipient chromosome homologs (designated N^D^ and N^R^), but not from the translocation chromosomes, were used as positive controls. The primers are listed in Table S3. Genomic DNA from *T* progeny can PCR amplify across the A, B, and C breakpoints, but not with the N^D^ and N^R^ primers; DNA from *Dp* progeny can PCR amplify across B and C but not A, and also give products with the N^D^ (but not N^R^) primers, while DNA from *N* does not amplify with primers for A, B, or C, but can give products with the N^D^ and N^R^ primers.

### Determining breakpoint junction sequences of T(V > VI)UK3-41

The *N. crassa* insertional translocation *T(VR > VIL)UK3-41* translocates 1,879,356 bp, bearing 490 genes, from chromosome 5R to 6L (Perkins 1997). Its translocated segment is larger than all the four previously introgressed translocations combined (Singh 2010; Singh *et al.* 2010). Its A breakpoint junction was previously determined (Genbank accession number HM573450), whereas the B and C breakpoint junction sequences were determined in this work (Genbank accession numbers KU554720 and KU599833). Genomic DNA of the *T(UK3-41)* strain was digested with *Hae*III, self-ligated, and the ligation product used as template in inverse PCR reactions with the primers listed in Table S4.

### Introgression of T(V > VI)UK3-41

Crosses between standard *N. crassa* strains of the Oak Ridge (OR) background and *N. tetrasperma* strains of the 85 background are almost completely sterile (Perkins 1997; Giri *et al.* 2015). However, both OR *A* and 85 *A* can cross with the *N. crassa*/*N. tetrasperma* hybrid strain *C4,T4 a* and produce viable progeny, thus allowing the *C4,T4 a* strain to be used as a bridging strain for the initial introgression crosses.

The *T(UK3-41)A* strain was crossed with *C4,T4 a*, and *T* progeny from these crosses (designated *T^1xC4T4^*) were distinguished from their *Dp* and *N* siblings by PCR with A and C breakpoint junction-specific primers. Nominally, 50% of the genome of *T^1xC4T4^* progeny is derived from the *C4,T4 a* parent. The *T^1xC4T4^A* progeny strains were crossed with *C4,T4 a* to obtain the *T^2xC4T4^* progeny in a like manner. Crosses of *T^2xC4T4^* with the opposite mating type derivative of strain 85 were productive, and their *T* progeny were designated as *T^1x85^*. Likewise, *T^1x85^* × 85 yielded *T^2x85^*, etc. Ordinarily, after two to three iterations of the crosses with 85, one can recover progeny ascospores that produce mycelia of dual mating specificity characteristic of *N. tetrasperma* (Giri *et al.* 2015). That is, the resulting mycelium could cross with both 85*A* and *a*, and it could also undergo a self-cross. However, as documented in the *Results* section, deviation from the expected Mendelian ratio in the progeny caused us to run out of *T(UK3-41)* progeny for additional crosses by the f_4_ generation.

### Data availability

Accession numbers of nucleotide sequences determined in this work: KU554720 and KU599833 (Genbank). 

## Results

### The max-4^-^ phenotype is not caused by a recessive mutation

The *T(IBj5)^Nt^ a* × *E A* and *T(B362i)^Nt^ A* × *E a* crosses showed the max-4^-^ phenotype (*i.e.*, no asci with > 4 black ascospores), whereas the *T(EB4)^Nt^ a* × *E A* and *T(UK14-1)^Nt^ A* × *E a* crosses produced asci with 8B:0W and 6B:2W ascospores (Giri *et al.* 2015). To account for these findings, it was suggested that the *E*, *T(IBj5)^Nt^* and *T(B362i)^Nt^* strains, but not *T(EB4)*^Nt^, *T(UK14-1)*^Nt^, 85 *A* and 85 *a*, contain a recessive mutation whose homozygosity induces the max-4^-^ phenotype. To test this hypothesis, we screened 103 and 58 f_1_ progeny from *E A* × 85 *a* and *E a* × 85 *A* and found, respectively, 57 and 21 homokaryons (*i.e.*, self-sterile), crossed them with *T(IBj5)^Nt^ a* or *T(B362i)^Nt^ A*, and examined the crosses for the max-4^-^ phenotype. We expected that the progeny inheriting the *E*-derived recessive allele would show the max-4^-^ phenotype, and those inheriting the 85-derived wild-type allele would also produce some 8B:0W and 6B:2W asci. Unexpectedly, all 78 f_1_ progeny showed the max-4^-^ phenotype (data not shown). This is possible if the chromosome bearing the *E*-derived mutant allele also exerts meiotic drive relative to its 85-derived homolog. However, we were unable to easily find polymorphic markers between the *E* and 85 strains to test if such was indeed the case.

Instead, we found several polymorphic markers between the 85/*EA*/*Ea* strains on the one hand and the *N. tetrasperma* FGSC 2508*A*/FGSC 2509*a* strains on the other (see *Materials and Methods*). We verified that these markers showed independent segregation in the homokaryotic progeny from the crosses *Ea* × 2508*A* (Table 1) and *EA* × 2509*a* (Table 2). Further, crosses of *T(IBj5)^Nt^a* and *T(B362i)^Nt^A* with 2508*A* and 2509*a* produced mostly four-spored asci, but the rare eight-spored asci included some 8B:0W, 4B:4W, and 6B:2W types (Table 3). This showed that the presumptive recessive mutation for the max-4^-^ phenotype is absent from the 2508*A* and 2509*a* strains. However, all homokaryotic f_1_ progeny examined from *Ea* × 2508*A* (N = 14) and *EA* × 2509*a* (*N* = 20) showed the max-4^-^ phenotype in crosses with *T(IBj5)^Nt^ a* or *T(B362i)^Nt^ A* (data not shown). Given that all chromosomes segregated independently in the f_1_ progeny, these results are incompatible with the idea that the max-4^-^ phenotype segregates with a specific chromosome from *EA* or *Ea*. Thus, the hypothesis that the max-4^-^ phenotype is caused by homozygosity for a recessive mutation is not tenable.

**Table 1:** Segregation of chromosomes in self-sterile progeny from the cross of *Ea* with 2508*A*

2508 *A* × *E a* (*n* = 28)													
Chromosome													
		**2**		**3**		**4**		**5**		**6**		**7**	
Chromosome	1	7	12	10	9	12	7	9	10	4	15	11	8
		3	6	3	6	6	3	3	6	3	6	5	4
	2			5	6	8	3	3	8	2	9	5	6
				8	9	10	7	9	8	5	12	11	6
	3					8	5	3	10	3	10	6	7
						10	5	9	6	4	11	10	5
	4							7	11	5	13	10	8
								5	5	2	8	6	4
	5			*EE*	*E+*					4	8	6	6
				*+E*	*++*					3	13	10	6
	6											5	2
												11	10

Each set of four figures in a column × row intersection cell presents the number of segregants with the parental genotypes in the upper left (*E^x^E^y^*) to lower right (*+^x^+^y^*) diagonal, and those with the recombinant genotypes in the upper right (*E^x^+^y^*) to lower left (*+^x^E^y^*) diagonal. The x and y superscripts indicate the chromosome bearing the respective marker alleles, where chromosome number x < than chromosome number y. The table provided by Perkins (1994) gives the smallest numerical ratios of parental and recombinant segregant numbers showing deviation in one direction from 1:1 at the 1% significance level, which is 21:7 (for *n* = 28). For all the markers, the parental to recombinants segregant ratios are smaller, showing that they do not differ from 1:1. This shows that in, all cases, the numbers of parental and recombinants are comparable, which suggests independent segregation of all seven chromosomes.

**Table 2:** Segregation of chromosomes in self-sterile progeny from the cross of *EA* with 2509*a*

2509 *a* × *E A* (*n* = 24)													
Chromosome													
		**2**		**3**		**4**		**5**		**6**		**7**	
Chromosome	1	5	10	7	8	8	7	7	8	5	10	5	10
		3	6	5	4	6	3	3	6	6	3	1	8
	2			4	4	4	4	5	3	5	3	3	5
				8	8	10	6	5	11	6	10	3	13
	3					9	3	2	10	4	8	2	10
						5	7	8	4	7	5	4	8
	4							4	6	6	8	4	10
								10	4	5	5	2	8
	5									5	5	3	7
										6	8	3	11
	6											3	8
												3	10

Each set of four figures in a column × row intersection cell presents the number of segregants with the parental genotypes in the upper left to lower right diagonal, and those with the recombinant genotypes in the upper right to lower left diagonal, using the same conventions used in Table 1. The table provided by Perkins (1994) gives the smallest numerical ratios of parental and recombinant segregant numbers showing deviation in one direction from 1:1 at the 1% significance level, which is 19:5 (for *n* = 24). For all the markers, the parental to recombinants segregant ratios are smaller, showing that they do not differ from 1:1. This shows that, in all cases, the numbers of parental and recombinants are comparable, which suggests independent segregation of all seven chromosomes.

**Table 3 T:** *(IBj5)^Nt^a* and *T(B362i)^Nt^A* crossed with the normal sequence FGSC 2508*A* or FGSC 2509*a* strains yield eight-spored asci that include the 8B:0W and 6B:2W types

Cross	*N*	Ascus Types (%)*^a^*								
		4	5	6	7	8:0	6:2	4:4	2:6	0:8
*T (IBj5)^Nt^ a* × *2508 A*	215	24	26	29	14	0	1	2	1	2
*T (B362i)^Nt^ A* × *2509 a*	316	64	20	10	3	2	1	1	0	0

*N*, number of asci examined on water agar.

a Percentages of 4-, 5-, 6-, 7-, and 8-spore ascus types are indicated. The 8-spored asci are further identified as 8:0, 6:2, 4:4, 2:6, and 0:8 types based on black: white ascospore numbers.

Howe and Haysman (1966) had reported that the *E* mutation segregates with chromosome 6. Since this was easy to confirm, it seemed worthwhile to do so. We crossed the homokaryotic f_1_ progeny from *Ea* × 2508*A* and *EA* × 2509*a* with opposite mating type derivatives of strain 85 and examined the progeny asci for the eight- or four-spore phenotype. Our results (Table S5) confirmed that *E* segregates with chromosome 6.

### Transmission ratio distortion in the progeny from some T^Nt^ × N crosses

Self-sterile progeny (*i.e.*, mating type homokaryons) were identified from crosses of the *T^Nt^* strains with *E* and 85 strains of the opposite mating type, and their *T*, *N*, or *Dp* genotype was determined by PCR. As expected, and as can be seen in the results summarized in Table 4, more self-sterile progeny were produced from crosses with the *E* strains. Additionally, we found three different phenotypes: the crosses of *T(EB4)^Nt^a* with the *N A* strains produced comparable numbers of *T*, *N*, and *Dp* progeny (*T* = *N* = *Dp*), whereas those of *T(IBj5)^Nt^a*, and *T(B362i)^Nt^A* × 85 *a*, produced far fewer *T* and *N* progeny than *Dp* type (*T*, *N* << *Dp*), and *T(B362i)^Nt^A* × *E a* produced *T* << *N*, *Dp* (Table 4). The B junction of *T(UK14-1)^Nt^*, a *QT*, is not yet defined. Consequently, only the *T* progeny from *T(UK14-1)^Nt^ A* × *N a* could be identified by their A breakpoint junction, whereas *Dp* and *N* were not distinguishable (Table 4). *Dp* segregants have only the B breakpoint and not A, whereas *N* segregants have neither.

**Table 4:** Homokaryotic progeny from some *T^Nt^* × *N* crosses show deviation from the Mendelian ratio

Parental Strains*^a^*		Progeny	Homokaryons			ALT: ADJ*^b^*	Phenotype
*T^Nt^*	*N*	*N* (Self-Sterile)	*T*	*N*	*Dp*		
*EB4 a*	85	129 (31)	11	11	9	22:18	*T*=*N*=*Dp*
	*E*	60 (40*^c^*)	7	15	17	22:34	*T*=*N*=*Dp*
*IBj5 a*	85	133 (25)	1	1	23	2:46**	*T*, *N*<< *Dp*
	*E*	82 (33*^d^*)	0	2	28	2:56**	*T*, *N*<< *Dp*
*UK14-1 A**^e^*	85	27 (17)	2	15		—	—
	*E*	30 (21)	4	17		—	—
*B362i A*	85	163 (59*^f^*)	0	3	48	3:96**	*T*, *N*<< *Dp*
	*E*	77 (61*^g^*)	0	27	14	27:28	*T*<< *N*, *Dp*

The number of progeny that were self-sterile is given in parentheses. The succeeding columns give numbers with the *T*, *N*, and *Dp* genotype, as determined by PCR. The *T*, *N*, and *Dp* columns exclude putative heterokaryons whose constituent nuclei have the same mating type. *P* < 0.01 **. ALT, alternate segregation; ADJ, adjacent-1 segregation; *N*, number of progeny examined; PCR, polymerase chain reaction.

a *T^Nt^* strains were crossed with opposite mating type derivative of strain 85 and *E*.

b ALT produces *T* and *N* progeny, whereas ADJ yields *Dp* and *Df*. Since *Df* progeny are inviable, we doubled the number of *Dp* progeny to estimate the ADJ-derived number. The chi-square test was used to establish that the ALT:ADJ ratio shows significant deviation in one direction from 1:1, and defines the phenotype *T*, *N*<< *Dp*.

c One progeny did not amplify for any translocation junction or for “normal” sequence on the donor and recipient chromosomes. It may be *N* type, in which the “normal A” and “normal B” primer binding sites are mutated.

d Three progeny amplified for junctions B and C, but not junction A or “normal A.” They may be *T* or *Dp* type, whose A or “normal A” primer binding site is mutated.

e *N* and *Dp* types were indistinguishable (see text for details).

f Five progeny amplified for junctions A, B, and C, and for “normal” sequences on the donor and recipient chromosomes, suggesting they are [*T^Nt^* + *N*] or [*Dp* + *Df*] heterokaryons; two amplified for “normal” sequences on the donor and recipient chromosome and for junctions B and C, but not A, suggesting they are [*N* + *Dp*]. One amplified only for “normal A” sequence but not for “normal” on the recipient chromosome. It might be *N* type, whose “normal B” primer-binding sequence is mutated.

g 19 progeny amplified for “normal” sequences on the donor and recipient chromosome, and junctions B and C, but not A, suggesting the [*N* + *Dp*] genotype, and one amplified only for junctions B and C and not for any normal sequences. It might be [*N* + *Dp*] or [*T^Nt^* + *Dp*] whose “normal A” or A primer-binding sequence is mutated.

Our results show evidence for transmission ratio distortion (*i.e.*, deviation from *T* = *N* = *Dp*) in the homokaryotic progeny from crosses of *E* or 85 strains with *T^Nt^* strains which gave the max-4^-^ phenotype in *T^Nt^* × *E* crosses. The distortion appeared to be specific to the homokaryotic progeny because [*Dp* + *Df*] and [*T^Nt^* + *N*] heterokaryotic (self-fertile) progeny did not show comparably distorted ratios. The [*Dp* + *Df*]/[*T^Nt^* + *N*] ratio was 3/3 and 7/3 in crosses with *T(IBj5)^Nt^* and *T(B362i)^Nt^* (which showed the max-4^-^ phenotype in crosses with *E*), and 4/4 and 2/3 in crosses with *T(EB4)^Nt^* and *T(UK14-1)^Nt^* (which did not) (Giri *et al.* 2015).

### Ascospores from 8B:0W asci from T^Nt^ × 85 have unexpected genotypes

Although the crosses of *T(IBj5)^Nt^a* and *T(B362i)^Nt^A* with 85*A*/85*a* or 2508*A*/2509*a* produced rare 8B:0W and 6B:2W asci, hardly any *T* progeny were obtained from the crosses of these *T^Nt^* strains with 85 (Table 4). To further investigate this anomaly, we collected asci from *T(IBj5)^Nt^a* × 85*A* and *T(B362i)^Nt^A* × 85*a* onto water agar. The majority of asci from these crosses were four- or five-spored, but we could also pick the rare seven- and eight-spored asci (Table 5), and we used PCR to determine the genotype of the cultures produced from their black ascospores.

**Table 5:** Ascus types from *T^Nt^* × 85

Cross	*N*	Ascus Types*^a^* (%)								
		4	5	6	7	8:0	6:2	4:4	2:6	0:8
*T (IBj5)^Nt^ a* × 85 *A*	834	66	21	7	5	0.4	0.2	0.1	0.1	0.4
*T (B362i)^Nt^ A* × 85 *a*	315	48	35	4	2	5	3	3	0.3	0

*N*, number of asci examined on water agar.

a Percentages of 4-, 5-, 6-, 7-, and 8-spored ascus types are indicated. The 8-spored asci are further identified as 8:0, 6:2, 4:4, 2:6, and 0:8 types based on black: white ascospore numbers.

Ten eight-spored asci were obtained from the cross *T(IBj5)^Nt^ a* × 85 *A*, including two 8B:0W, one 7B:1W, two 5B:3W, one 4B:4W, one 2B:6W, and three 0B:8W types. Two progeny were obtained from the 4B:4W ascus, and both amplified for junction B and normal A indicating that they had *Dp* genotype; one was *mat A* and the other was *mat a*. This was consistent with the expectation that 4B:4W asci yield only *Dp* progeny. Three progeny were obtained from one 8B:0W and one 7B:1W ascus. One appeared to be a [*T^Nt^* + *N*] or [*Dp* + *Df*] heterokaryon bearing both *mat A* and *mat a* nuclei, one was *Dp a*, and one was *T A*. Normally, one does not expect heterokaryotic and *Dp* genotypes in progeny ascospores from 8B:0W and 7B:1W asci. Additionally, we examined six progeny from two seven-spored asci (one 7B:0W + one 5B:2W). Three were *Dp a*, one was *N a*, and two were [*Dp* + *N*] heterokaryons possessing both *mat A* and *mat a* nuclei. This result was also unexpected because one expects to find three *Df A* type progeny complementary to the three *Dp a* types, but the two asci together contained only two white ascospores. These results show that. while the progeny from the 4B:4W asci from *T(IBj5)^Nt^ a* × 85 *A* have the expected *Dp* genotype, those from asci containing > 4 viable ascospores often display unexpected genotypes.

We obtained 36 eight-spored asci from the *T(B362i)^Nt^A* × 85*a* cross, including 10 8B:0W, six 7B:1W, five 6B:2W, four 5B:3W, eight 4B:4W, two 3B:5W, and one 2B:6W types. All three progeny examined from the 4B:4W asci had the expected *Dp* genotype. In contrast, none of the 17 progeny examined from the 8B:0W asci were *T* or *N* type. Six appeared to be [*T* + *N*] or [*Dp* + *Df*] heterokaryons. Four of them were mating type homokaryons, whereas two had *mat A* and *mat a* nuclei. Two progeny appeared to be [*Dp* + *N*] heterokaryons, and nine appeared to have the *Dp* genotype. None of these genotypes are expected in ascospores from 8B:0W asci. From the 6B:2W asci, one progeny appeared to be a [*T* + *N*] or [*Dp* + *Df*] heterokaryon, two appeared to be [*Dp* + *N*] heterokaryons, ten were *Dp* type, and one was *N* type. Heterokaryotic genotypes are not expected in ascospores from 6B:2W asci. Again, these results show that although progeny from the 4B:4W asci from the *T(B362i)^Nt^A* × *85a* cross have the expected *Dp* genotype, those from the 8B:0W and 6B:2W asci often display unexpected genotypes.

### Introgressing T(V > VI)UK3-41

*Hae*III-digested genomic DNA of the *T(VR > VIL)UK3-41A* strain [henceforth, *T(UK3-41) A*] was self-ligated, and used as a template for inverse PCR with primers complementary to sequences within the translocated segment. In this way, we retrieved the adjoining sequence on the recipient chromosome and thus defined the B and C breakpoint junctions (respective Genbank accession numbers KU599833 and KU554720). The flanking sequence of the B junction had a *Hae*III site located merely 2 bp proximal to the breakpoint junction. Obtaining only 2 bp of proximal flanking sequence from the recipient chromosome was inadequate to design a primer to PCR amplify across the breakpoint junction. On normal sequence chromosome 6L, a 15,882 bp AT-rich (70.5%) sequence containing only a few 4 bp-cutting restriction sites separates the C breakpoint locus from its closest proximal ORF (ncu 07116). These restriction enzymes did not have any convenient sites within the UK3-41 translocated segment, making it difficult to design alternative inverse PCR strategies to retrieve additional sequences proximal to the translocated segment. Therefore, the B breakpoint remains undetermined. Figure S3 updates the breakpoints of 12 *Dp*-generating translocations on the *N. crassa* genome sequence.

**Figure 3:**
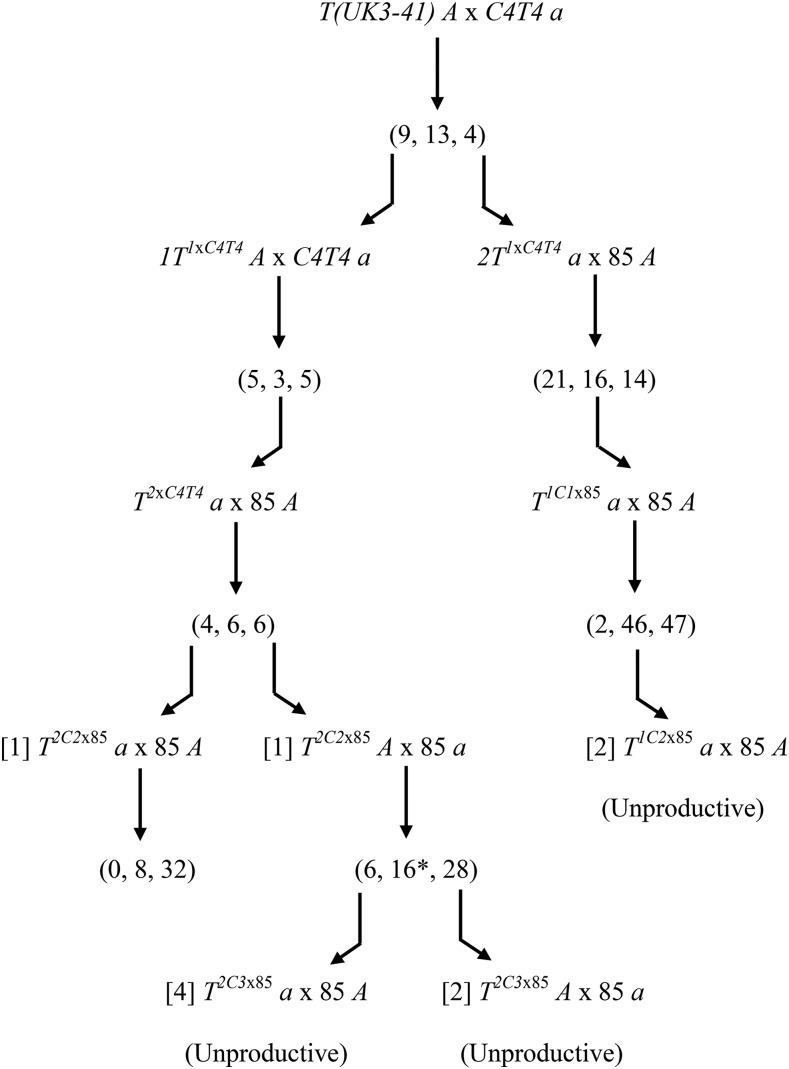
Attempt to introgress *T(UK3-41)* into *N. tetrasperma*. *N. crassa* strain *T(UK3-41) A* was crossed with the C4T4 *a* hybrid strain. PCR with breakpoint junction-specific primers was done to identify the progeny types, and the number of translocation, normal sequence, and duplication types is indicated in the sequence (*T*, *N*, *Dp*). Bent arrows represent the *T* progeny used for the next round of crosses (*e.g.*, *1T^1xC4T4^A*, *2T^1xC4T4^ a*). *1T^1xC4T4^A* × *C4T4 a* yielded the strain *T^2xC4T4^ a*, which was productive in crosses with *N. tetrasperma* strain 85 *A*. Of the 4 *T^2C2^*^x85^ progeny, two were unproductive in crosses, one (*T^2C2^*^x85^ *a*) was productive in the cross with 85 *A*, and its cross produced 0 *T* progeny; its sibling strain *2T^1xC4T4^ a* was productive in the cross with 85 *A*, producing six *T* progeny strains (4 *T^2C3^*^x85^ *a* and 2 *T^2C3^*^x85^ *A*) that gave unproductive crosses with 85 *A* or 85 *a*. One of the 16 *N* type progeny (asterisk) from *T^2C2^*^x85^ *A* × 85 *a* was a self-fertile [*mat A* + *mat a*] heterokaryon. The crosses of two *T^1C2^*^x85^ *a* strains with 85 *A* also were unproductive. PCR, polymerase chain reaction.

Knowledge of the A and C breakpoint junctions of *T(UK3-41)* allowed us to distinguish the *T*, *Dp*, and *N* progeny from *T(UK3-41)* × *N* crosses. Therefore, we attempted to introgress this translocation into *N. tetrasperma* (Figure 3). The initial crosses of *T(UK3-41)* strains with C4T4 *a* and 85 *A* showed the *T* = *N* = *Dp* phenotype, but subsequent to the first productive *T a* × 85 *A* crosses the crosses of the *T* progeny with 85 *A* or 85 *a* gave the *T* << *N*, *Dp* phenotype (see above). One introgression line (left in Figure 3), produced no *T* progeny in the f_4_, and another two (middle and right in Figure 3) gave six f_4_ and two f_3_ *T* progeny that were unproductive in subsequent crosses with strain 85 derivatives of opposite mating type. Thus, transmission ratio distortion (TRD), similar to that associated with the max-4^-^ phenotype, precluded our obtaining sufficient numbers of *T* progeny. Defining the TRD-inducing gene will help us to identify early generation translocation progeny from which it is absent, and facilitate future introgression efforts.

## Discussion

In this study we sought to further explore the unusual phenotype exhibited by some *T^Nt^* × *E* crosses, wherein no asci contain more than four black ascospores (Giri *et al.* 2015). We now call this the max-4^-^ phenotype. Our results suggest that the max-4^-^ phenotype is not caused by homozygosity for a recessive mutation. Instead, we now suggest that one or more BDMI between *N. crassa* and *N. tetrasperma* genes has different consequences on homokaryotic ascospores generated following ALT *vs.* ADJ. Crosses of the *T^Nt^* strains with the normal-sequence 85 *A*, 85 *a*, *E A*, and *E a* strains of *N. tetrasperma* showed one of three phenotypes, *viz*., *T* = *N* = *Dp*; *T*, *N* << *Dp*; or *T* << *N*, *Dp*. In contrast, *N. crassa T* × *N* crosses show only the *T* = *N* = *Dp* phenotype (Perkins 1997; Giri *et al.* 2015). Transmission ratio distortion, as seen in *T*, *N* << *Dp* and *T* << *N*, *Dp* was evident only in crosses with *T^Nt^* strains that induced the max-4^-^ phenotype in crosses with *E*. This suggested that the max-4^-^ phenotype and the transmission ratio distortion are related. The *T* and *N* type progeny are generated by ALT, whereas ADJ produces *Dp* and *Df*. Differential recovery of the products of ALT and ADJ, as in *T*, *N* << *Dp*, represents a novel type of meiotic drive, one detectable only in crosses heterozygous for insertional and quasi-terminal translocations, because ALT and ADJ are not evident in *N* × *N* and *Dp* × *N* crosses, and all the products from ADJ are inviable in *RT* × *N* crosses. We do not think ALT is less frequent than ADJ in our system, because that would not explain the production of 0B:8W and 2B:6W asci.

[*T^Nt^* + *N*] and [*Dp* + *Df*] asci contain the same genes but distribute them differently in the *mat A* and *mat a* nuclei. Any model must translate this difference into the differential ascospore viability seen in the *T*, *N* << *Dp* and *T* << *N*, *Dp* phenotypes. One model (model 1) is that the *N. crassa*-derived *T* recipient chromosome and the *N. tetrasperma*-derived homolog of the *T* donor chromosome carry meiotic drive elements that induce inviability in progeny that do not inherit them. Meiotic drive elements are selfish genes that skew the 1:1 Mendelian segregation ratio to their own advantage and their presence, in turn, imposes selection for unlinked suppressors that restore the Mendelian ratio. Reproductively isolated taxa such as *N. crassa* and *N. tetrasperma* may have accumulated distinct drivers and suppressors that might get separated in the hybrid *T^Nt^* strains, and allow drive to surface in their crosses (Orr *et al.* 2007; Zanders *et al.* 2014). ALT would segregate the meiotic drive elements into the *T* and *N* progeny, thus rendering both inviable, whereas ADJ would segregate both elements into the *Dp* progeny, resulting in their survival. In the original *T^Nt^* strain, isolated as a self-sterile conidial derivative from a [*T^Nt^* + *N*] heterokaryon, the opposite mating type nuclei each contained a meiotic drive element, thus rescuing the heterokaryotic ascospore. A similar model was proposed to explain why hybrids of *Schizosaccharomyces* species *Schizosaccharomyces kambucha* and *S. pombe* yielded more aneuploid and diploid progeny than they did of either parental genotype (Zanders *et al.* 2014). Similarly, the meiotic drive spore-killer elements in *Neurospora* and *Podospora*, *Sk-2*, *Sk-3*, *Spok1*, and *Spok2*, trigger inviability in ascospores not inheriting them (Turner and Perkins 1979; Hammond *et al.* 2012; Grognet *et al.* 2014; Harvey *et al.* 2014). This model is constrained by the fact that it requires the meiotic drive elements to be correctly positioned in *T(IBj5)^Nt^* and *T(B362i)^Nt^*. It also does not readily account for the *T* << *N*, *Dp* phenotype. Chromosome 4 is the donor chromosome in *T(B362i)^Nt^*, and if a meiotic drive element was present on chromosome 4 of the *E* strain, then its segregation in *T(B362i)^Nt^A* × *E a* could make the *T* progeny that do not inherit it unviable, whereas the *N* and *Dp* progeny that do would survive. However, chromosome 4 is the recipient chromosome in *T(IBj5)^Nt^*; therefore, the model predicts inviability of the *Dp* progeny from *T(IBj5)^Nt^a* × *E A*, which is clearly not the case.

Model 2 invokes BDMI between *N. crassa* and *N. tetrasperma* genes giving rise to, say, an insufficiency for an ascospore maturation product in asci from *T^Nt^* × *N* crosses. Only four viable ascospores (hetero- or homokaryotic) are made following ADJ, whereas four (all heterokaryotic) to eight (all homokaryotic) viable ascospores can be produced following ALT. The generation of > 4 viable ascospores might precipitate a “tragedy of the commons,” wherein no ascospore receives a sufficient amount of the maturation factor. This would specifically disfavor the ALT-derived homokaryotic progeny.

How might one explain the *T* << *N*, *Dp* phenotype? One possibility is that the homokaryotic *N* cultures are secondarily produced via loss of *T* nuclei from heterokaryotic [*T^Nt^* + *N*] germlings, thus masking the *T*, *N* << *Dp* phenotype in the *T(B362i)^Nt^A* × *E a* cross. The *T* genotype has more *N. crassa*-derived sequences than *N*, especially on the translocation donor and recipient chromosomes. Conceivably, a second BDMI could select against the *T* nuclei during vegetative growth. However, we do not rule out the possibility that the second BDMI affects the viability of *T* ascospores in [*T^Nt^* + *N*] asci to produce an effectively four-spored [*T^lethal^* + *N*] ascus. Thus, both the *T*, *N* << *Dp* and *T* << *N*, *Dp* phenotypes can potentially be explained by BDMIs that generate the max-4^-^ phenotype. *T^Nt^* strains (*e.g.*, *T(EB4)^Nt^ a*), from which the *N. crassa*-derived meiotic drive element or BDMI gene(s) is absent, are not expected to show transmission ratio distortion in crosses with *N* strains.

The production of only [*T^Nt^* + *N*], [*Dp* + *Df*], and *Dp* progeny would act to impede gene flow between *N. crassa* and *N. tetrasperma* because heterokaryotic strains tend not to out-cross as females (Bistis 1996), and meiotic silencing by unpaired DNA (MSUD) renders *Dp* × *N* crosses barren (Shiu *et al.* 2001). MSUD is triggered by the transcription of “aberrant RNA” from improper pairing of *Dp*-borne genes in meiosis, and its processing into MSUD-associated small interfering RNA (masiRNA) used by a silencing complex to identify and degrade complementary mRNA as it exits the nucleus (Hammond *et al.* 2013).

Finally, our results revealed that the rare 8B:0W and 6B:2W asci from the *T^Nt^A* × 85 crosses can mask the max-4^-^ phenotype. Analysis of progeny from such asci showed that they include heterokaryons, or other genotypes incommensurate with the supposition that the ascospores are initially uninucleate and receive one of the eight nuclei made following the postmeiotic mitosis. It is possible that in a subset of [*Dp* + *Df*] or [*Dp* + *N*] asci the nuclei might undergo additional mitoses beyond the first postmeiotic mitosis before being partitioned into the eight ascospores. This is a novel and unprecedented finding.

## Supplementary Material

Supplemental Material
