# Supplementary material for: Crosses Heterozygous for Hybrid Neurospora Translocation Strains Show Transmission Ratio Distortion Disfavoring Homokaryotic Ascospores Made Following Alternate Segregation
Source: G3 (Bethesda). 2016 Jun 17;6(8):2593–600. doi: 10.1534/g3.116.030627 (PMC4978912; doi:10.1534/g3.116.030627)
Supplement: Supplemental Material [file supp_6_8_2593__index.html]

Crosses Heterozygous for Hybrid Neurospora Translocation Strains Show Transmission Ratio Distortion Disfavoring Homokaryotic Ascospores Made Following Alternate Segregation — Supplemental Material 

# Crosses Heterozygous for Hybrid *Neurospora* Translocation Strains Show Transmission Ratio Distortion Disfavoring Homokaryotic Ascospores Made Following Alternate Segregation

## Supplemental Material for Giri, Rekha, and Kasbekar, 2016

**Files in this Data Supplement:**

- Figure S1 - Alternate (ALT) and adjacent-1 (ADJ) segregation in a normal sequence (*N*) by reciprocal translocation (*RT*) cross. (.pdf, 104 KB)
- Figure S2 - Chromosome positions of markers polymorphic between the *N. tetrasperma* 85/*EA*/*Ea* and FGSC 2508*A*/FGSC 2509*a* strains. (.pdf, 112 KB)
- Figure S3 - Breakpoints of *Dp*-generating translocations mapped on the *N. crassa* genome sequence. (.pdf, 288 KB)
- Table S1 - Neurospora strains used in this study. (.pdf, 13 KB)
- Table S2 - PCR primers and restriction enzymes used to obtain markers polymorphic between the *EA*/*Ea* and FGSC 2508 *A*/FGSC 2509 *a* strains. (.pdf, 17 KB)
- Table S3 - Primers used in PCR to identify *T*, *N*, and *Dp* progeny. (.pdf, 16 KB)
- Table S4 - Primers used in inverse PCR spanning the B and C breakpoint junctions of *T*(*UK3-41*). (.pdf, 156 KB)
- Table S5 - The *Eight-spore* mutation (*E*) segregates with chromosome 6. (.pdf, 27 KB)
