# Supplementary material for: Crosses Heterozygous for Hybrid Neurospora Translocation Strains Show Transmission Ratio Distortion Disfavoring Homokaryotic Ascospores Made Following Alternate Segregation
Source: G3 (Bethesda). 2016 Jun 17;6(8):2593–600. doi: 10.1534/g3.116.030627 (PMC4978912; doi:10.1534/g3.116.030627)
Supplement: Supplemental Material [file supp_g3.116.030627_FigureS1.pdf]

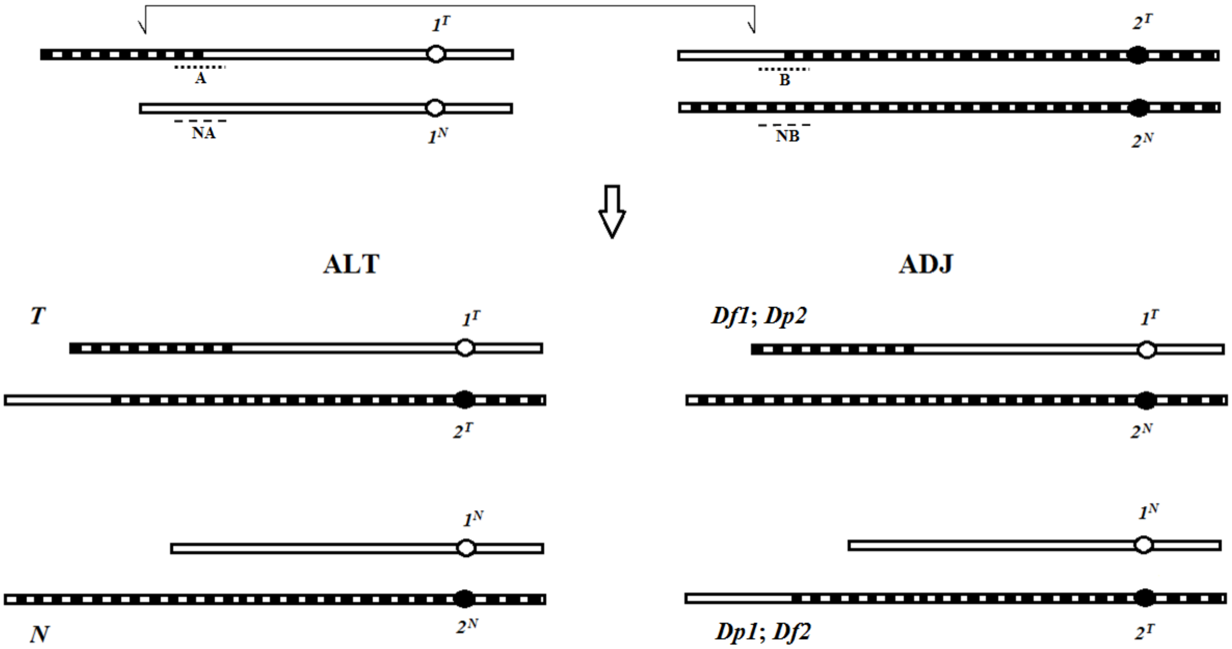

Figure S1. Alternate (ALT) and adjacent-1 (ADJ) segregation in a normal sequence (*N*) by reciprocal translocation (*RT*) cross. 1^T and 2^T designate the two chromosomes of the *RT* whose terminal segments are reciprocally exchanged, and 1^N and 2^N represent their normal sequence homologs. The A and B breakpoint junctions are indicated by the dotted lines, and dashed lines NA and NB indicate segments in the normal sequence homologues that are disrupted in the translocation chromosomes. In alternate segregation (ALT, lower left) 1^T and 2^T segregate to one spindle pole, and 1^N and 2^N to the other. Subsequently, meiosis II and post-meiotic mitosis generate eight parental-type nuclei, viz. 4 *RT* + 4 *N*. In adjacent-1 segregation (ADJ, lower right), 1^T and 2^N segregate to one pole and 1^N and 2^T to the other, to produce two non-parental genotypes *Dp2*; *Df1* or *Dp1*; *Df2*. The deficiencies cause inviability, consequently all the progeny ascospores remain unpigmented and the asci are 0B:8W type. Since ALT and ADJ are equally likely, the number of 8B:0W asci from ALT equals the number of 0B:8W asci from ADJ, hence obtaining 8B:0W = 0B:8W is a characteristic of *RT* x *N*.
