# Supplementary material for: Crosses Heterozygous for Hybrid Neurospora Translocation Strains Show Transmission Ratio Distortion Disfavoring Homokaryotic Ascospores Made Following Alternate Segregation
Source: G3 (Bethesda). 2016 Jun 17;6(8):2593–600. doi: 10.1534/g3.116.030627 (PMC4978912; doi:10.1534/g3.116.030627)
Supplement: Supplemental Material [file supp_g3.116.030627_FigureS2.pdf]

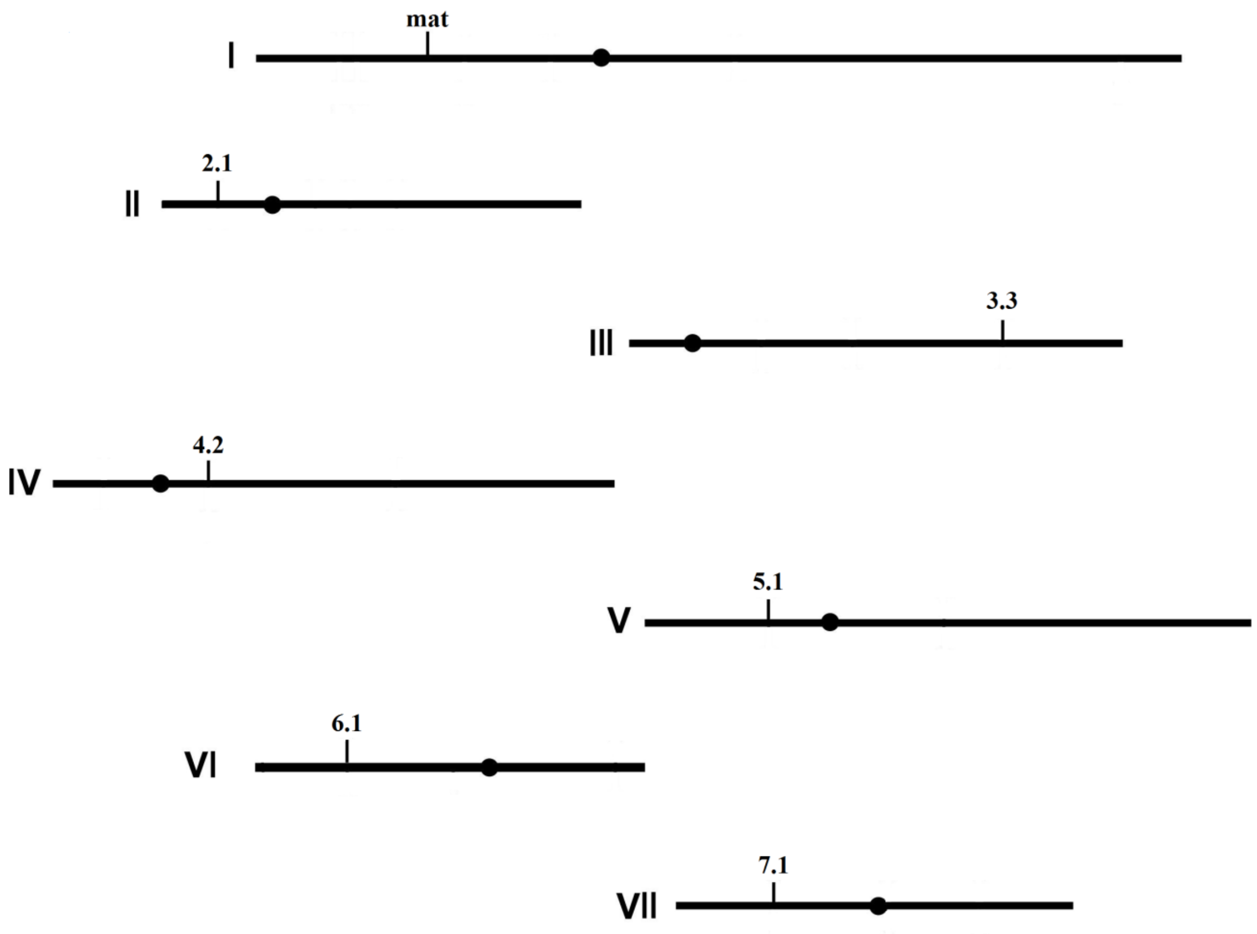

Figure S2. Chromosome positions of markers polymorphic between the *N. tetrasperma* 85/*EA*/*Ea* and FGSC 2508*A*/FGSC 2509*a* strains.
