# Supplementary material for: Crosses Heterozygous for Hybrid Neurospora Translocation Strains Show Transmission Ratio Distortion Disfavoring Homokaryotic Ascospores Made Following Alternate Segregation
Source: G3 (Bethesda). 2016 Jun 17;6(8):2593–600. doi: 10.1534/g3.116.030627 (PMC4978912; doi:10.1534/g3.116.030627)
Supplement: Supplemental Material [file supp_g3.116.030627_FigureS3.pdf]

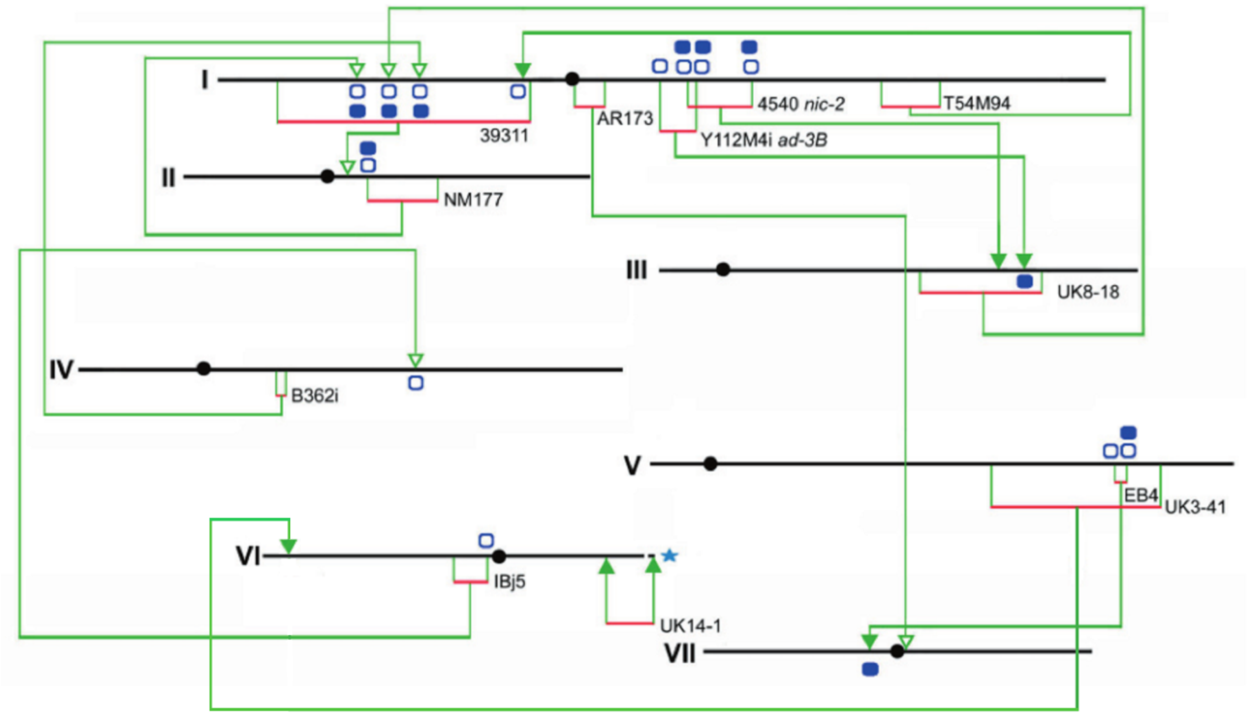

Figure S3. Breakpoints of *Dp*-generating translocations mapped on the *N. crassa* genome sequence. Black lines represent the sequenced chromosomes. Arrowheads indicate the insertion sites of the donor chromosome segments on the recipient chromosomes. Filled arrowheads indicate that the translocated segment is inserted non-inverted relative to the centromere, while open arrowheads signify segments that are inverted relative to the centromere. Open circles indicate genes disrupted by the translocations and filled circles indicate novel open reading frames. The break on the donor chromosome of the quasiterminal translocation *T(UK14-1)* is capped by sequence from the unassigned supercontig 10.9 (star), suggesting that supercontig 10.9 might be in distal LG VIL. The figure updates Figure 2 of Singh *et al*. (2010).
