# Supplementary material for: Crosses Heterozygous for Hybrid Neurospora Translocation Strains Show Transmission Ratio Distortion Disfavoring Homokaryotic Ascospores Made Following Alternate Segregation
Source: G3 (Bethesda). 2016 Jun 17;6(8):2593–600. doi: 10.1534/g3.116.030627 (PMC4978912; doi:10.1534/g3.116.030627)
Supplement: Supplemental Material [file supp_g3.116.030627_TableS1.pdf]

Table S1: Neurospora strains used in this study.

Strain	FGSC No.	Genotype	Reference
N. crassa			
T(UK3-41) A	6869	*T(VR>VIL)UK3-41, inl A*	1
N. tetrasperma			
85 *A*	1270	Wild type *A*	2
85 *a*	1271	Wild type *a*	2
E A	2783	*lwn; al(102),E A*	
E a	2784	*lwn; al(102),E a*	
2508	2508	Wild type *A*	
2509	2509	Wild type *a*	
T(EB4)^{Nt} *a*	25016	*T(VR>VIIL)EB4 A*	3
T(IBj5)^{Nt} *a*	25017	*T(VIL>IR)IBj5 cpc-1 A*	3
T(UK14-1)^{Nt} *A*	25018	*T(VIR>VL)UK14-1 A*	3
T(B362i)^{Nt} *A*	25019	*T(IVR>IL)B362i A*	3
**Hybrid**			
C4,T4 a	1778	*N. crassa* / *N. tetrasperma* hybrid strain *a*	4

[1]Perkins (1997), [2]Raju and Perkins (1991), [3] Giri *et al*. (2015), [4] Metzenberg and Ahlgren (1969).
