# Supplementary material for: Crosses Heterozygous for Hybrid Neurospora Translocation Strains Show Transmission Ratio Distortion Disfavoring Homokaryotic Ascospores Made Following Alternate Segregation
Source: G3 (Bethesda). 2016 Jun 17;6(8):2593–600. doi: 10.1534/g3.116.030627 (PMC4978912; doi:10.1534/g3.116.030627)
Supplement: Supplemental Material [file supp_g3.116.030627_TableS2.pdf]

Table S2. PCR primers and restriction enzymes used to obtain markers polymorphic between the *EA*/*Ea* and FGSC 2508 *A*/FGSC 2509 *a* strains.

Linkage group (Sequence ID)		Primers	Primer positions	Restriction enzyme
I<br>GL891382	Mat A	F: CAAGACGTTCGCCGACCTCGCTG<br>R: GGGTCATTGCTGGAGGCGTGTC	1762668-1762690+<br>1763501-1763522-	-
I<br>M54787	Mat a	F: CCAAGCCATTCACCGCCCAG<br>R: GCGCCTGCGAATGTCTTGAG	2932-2951+<br>3905-3924-	-
II<br>GL891305	2.1	F: ACCTATGGACTGGACGAGGA<br>R: CGAGACGGAGAATGGAGAAC	3673576-3673595+<br>3675001-3675018-	*Mbo*I
III<br>GL891304	3.3	F: GCTGTATGCTGGAGTGTTGG<br>R: ACGGAAGTTGCTGTCTGCTT	3803885-3803904+<br>3805509-3805528-	*Mse*I
IV<br>GL891303	4.2	F: GGACTGACTCGCTGTGTGAG<br>R: GGGCACAGGTCGTGGAAATAG	1532192-1532211+<br>1533299-1533319-	*Hpa*II
V<br>GL891302	5.1	F: CGGTGTCGCTATGAGAGGAT<br>R: TGGTGGTGGTGTCAGTCAGT	4904923-4904942-<br>4903917-4903936+	*Sau*3AI/*Hae*III
VI<br>GL891307	6.1	F: ACATAAGCAGCGAACCGAAC<br>R: GCCGAGCGATAACACACACT	1008619-1008638+<br>1010388-1010407-	*Hpa*II/*Hae*III
VII<br>GL891306	7.1	F: CGCTATGCTCCGACTCTTCT<br>R: CACGCACACCTTGACAGTTC	2924512-2924531-<br>2922988-2923007+	*Hae*III
