# Supplementary material for: Crosses Heterozygous for Hybrid Neurospora Translocation Strains Show Transmission Ratio Distortion Disfavoring Homokaryotic Ascospores Made Following Alternate Segregation
Source: G3 (Bethesda). 2016 Jun 17;6(8):2593–600. doi: 10.1534/g3.116.030627 (PMC4978912; doi:10.1534/g3.116.030627)
Supplement: Supplemental Material [file supp_g3.116.030627_TableS3.pdf]

Table S3: Primers used in PCR to identify *T*, *N*, and *Dp* progeny.

Translocation	Breakpoint		
	A	B	C
**UK3-41**	GTGGTCATGCTGGTGTGCCC	ND	GATCATGCTCAAGGAGGAAGGG
	GCGACAGCAAAGGGAAGCAC	ND	GGCCACTCTAAGGAATAGGAGACG
**EB4**	GCCGGTTTTGGAGCATCCATACACAGGG	GCGGGCGGCAAAGGCTGTT	TCCACACCAGAGGTCGTAG
	AAGTTGTAGCTACGCTGAAACACCAGATGACC	GGGCGGCTTCGGCAGTAAA	ATCAATTCGCGATGGAACGG
**IBj5**	CTCTCGCCCGACTAGGACTTC	GCTGTGACTCATACTTCCCCC	ND
	GTTGCCCTGCTTTCCGTGCG	GTTCGCTAGTGAGTGCGTTCC	ND
**UK14-1**	GGTAGGTAAGGAAGGTGCAATCG	ND	NA
	CGATGAAGAGAGGCCCAGTGAAGAC	ND	
**B362i**	ATAGTGGGAGCTGTCACAGGTTCCCTTG	AGCTCGAATCGCGAGGAGAG	TTCATCGAGACCGGCTGGAAG
	AAGTTGTAGCTACGCTGAAACACCAGATGACC	GTCTTCGGGCTTCAACCGAG	CGCGATGTCACCGACGAAAG

	N^D	N^R
**UK3-41**	ATGGCGTGGAGCGA ACATAC	ND
	GAGTGCGAGCGAGT GTCTC	ND
**EB4**	ACTAGTACGGCATCC CACCC	CCGTGCGCGCAAC TAAATTC
	TCCCTTTCCAACTCT CGTGC	TGAGAATCGAAAG GCGGGGG
**IBj5[1]**	GGCACAGTCAATAC CATCGC AGTATGATTTGGGTC AACGGC	
**B362i**	GTGGTGGTACGTGGT ACCTG	GCCATCTGAGTGT ACTCGCC
	GTTGAAGCTGCGAC ACGATG	CTTACTGTCTGCG ACGGTCC

NA = not applicable, ND = not determined

[1] We did not get any reliable primer-set for normal recipient chromosome of IBj5.
