# Supplementary material for: Crosses Heterozygous for Hybrid Neurospora Translocation Strains Show Transmission Ratio Distortion Disfavoring Homokaryotic Ascospores Made Following Alternate Segregation
Source: G3 (Bethesda). 2016 Jun 17;6(8):2593–600. doi: 10.1534/g3.116.030627 (PMC4978912; doi:10.1534/g3.116.030627)
Supplement: Supplemental Material [file supp_g3.116.030627_TableS4.pdf]

Table S4: Primers used in inverse PCR spanning the B and C breakpoint junctions of *T(UK3-41)*.

	Primers
Junction B	5’CGAACCGTGAACCCCTTTCTAGC
	5’TGGATGTTGTGGTTTGGCGACTC
Junction C	5’GCGGTACAGGTACTCGGGATTC
	5’CCGGGGTATCGCAATGTGATCC
