# Supplementary material for: Crosses Heterozygous for Hybrid Neurospora Translocation Strains Show Transmission Ratio Distortion Disfavoring Homokaryotic Ascospores Made Following Alternate Segregation
Source: G3 (Bethesda). 2016 Jun 17;6(8):2593–600. doi: 10.1534/g3.116.030627 (PMC4978912; doi:10.1534/g3.116.030627)
Supplement: Supplemental Material [file supp_g3.116.030627_TableS5.pdf]

Table S5: The *Eight-spore* mutation (*E*) segregates with chromosome 6.

		Chromosomes[1]													
		**1**		**2**		**3**		**4**		**5**		**6**		**7**	
		E	+	*E*	+	*E*	+	*E*	+	*E*	+	*E*	+	*E*	+
2508 *A* x *E a*	[2]**E**	7	11	2	6	4	8	7	9	6	6	7	0	7	9
(n = 25)	[2]**+**	2	5	7	10	5	8	2	7	3	10	2	16	2	7
2509 *a* x *E A*	**E**	6	9	5	3	4	8	5	9	5	5	10	1	3	3
(n = 24)	**+**	5	4	6	10	7	5	6	4	6	8	1	12	8	10

[1](*E* +) = marker alleles, respectively, from the *E* and FGSC 2508 or FGSC 2509 strains. [2]E and [2]+ = Eight spored (**E**) or wild-type (**+**) ascus phenotypes. Each set of four figures presents the number of segregants with the parental **E** phenotype and *E* genotype or **+** phenotype and + genotype in the upper left (**E***E*) - lower right (**+**+) diagonal, and the numbers with the recombinant **E** phenotype and + genotype or **+** phenotype and *E* genotype in the upper right (**E**+) -lower left (**+***E*) diagonal. The table provided by Perkins (1994) gives the smallest numerical ratios of parental and recombinant segregant numbers showing deviation in one direction from 1:1 at the 1% significance level. These are 19:5 (for n = 24) and 19:6 (for n = 25). This ratio is exceeded only for the chromosome 6 marker. For all other markers the parental to recombinants ratios are smaller, showing that they do not differ from 1:1. This shows that the *E* mutation segregates with chromosome 6.
